# Advancing Sino-Indian Cooperation to Combat Tropical Diseases

**DOI:** 10.1371/journal.pntd.0002204

**Published:** 2013-09-26

**Authors:** Peter Hotez, Sunit K. Singh, Xiao-Nong Zhou

**Affiliations:** 1 Department of Pediatrics and Molecular Virology and Microbiology, National School of Tropical Medicine, Baylor College of Medicine, Houston, Texas, United States of America; 2 Sabin Vaccine Institute and Texas Children's Hospital Center for Vaccine Development, Houston, Texas, United States of America; 3 James A. Baker III Institute for Public Policy, Rice University, Houston, Texas, United States of America; 4 Laboratory of Neurovirology and Inflammation Biology, Centre for Cellular and Molecular Biology (CCMB), Council of Scientific and Industrial Research (CSIR), Hyderabad, India; 5 National Institute of Parasitic Diseases, Chinese Center for Disease Control and Prevention, Shanghai, China

In 2005 India, Nepal, and Bangladesh signed a landmark agreement to eliminate visceral leishmaniasis in South Asia. There is an exciting opportunity for India and China to also engage in international science diplomacy for controlling or eliminating the major neglected tropical diseases in their two countries, and thereby reducing the global NTD burden by up to 40% or more.

*Why are we in India so embarrassed to recognise our own strengths, our achievements? We are such a great nation. We have so many amazing success stories but we refuse to acknowledge them.* —Abdul Kalam (former President of India) [1]

*It is a trite saying that one half the world knows not how the other lives. Who can say what sores might be healed, what hurt solved, were the doings of each half of the world's inhabitants understood and appreciated by the other.* —Mahatma Gandhi [2]

China and India each have a population of more than one billion people, and today the two nations combined account for more than one-third of the world's population (Table 1) [3]. China and India are also important emerging economies with a combined gross domestic product (GDP) of more than $7 trillion, a dollar amount exceeded only by the economic output of the United States [4]. However, at the same time almost 600 million Chinese and Indians live below the World Bank poverty figure of $1.25 per day, and together these two nations account for almost one-half of the world's “bottom billion”, i.e., the 1.29 billion people who live on essentially no money [5], [6].

**Table 1:** Socioeconomic aspects of China and India.

Feature	China	India	Combined	Comment
1. Population [3]	1.347 billion	1.210 billion	2.557 billion	n/a
2. Approximate % global population [3]	19.1%	17.2%	36.3%	n/a
3. Gross domestic product (GDP) in 2010 [4]	$5.7 trillion	$1.7 trillion	$7.4 trillion	n/a
4. GDP Rank [4]	2nd	9th	2nd	n/a
5. Approximate % of global GDP [4]	9.1%	2.7%	11.8%	n/a
6. Percentage of people living on less than $1.25 per day [5]	13.06%	32.67%	22% (calculated)	n/a
7. Approximate number of people living on less than $1.25 per day	176 million	395 million	571 million	Calculated by combining features 1 and 6
8. Approximate percentage of the global population that lives on less than $1.25 per day	13.6%	30.6%	44.3%	Based on 1.29 billion people living below the World Bank poverty figure of $1.25 per day [6]

There is an intimate link between poverty and disease, especially for the neglected tropical diseases (NTDs) that disproportionately strike the bottom billion [7]. With so many people living in extreme poverty it should be no surprise that both China and India are middle-income countries plagued with widespread infectious and neglected tropical diseases [8]–[12].

Shown in Table 2 is a summary of the major NTDs affecting both countries [8]–[34]. By our current estimates, China and India account for roughly one-fifth of the world's soil-transmitted helminth infections (e.g., ascariasis, trichuriasis, and hookworm) [9], [10], [12]–[14], while India accounts for more than 40% of the world's population requiring mass drug administration for lymphatic filariasis [15]. China also suffers from the greatest number of cases of liver fluke infection (caused by *Clonorchis sinensis*) and lung fluke infection (caused by *Paragonimus* spp.), and accounts for most of the world's disease burden from these two infections [9], [10], [16] as well as for more than 90% of the disease burden from alveolar echinococcosis [17]. Alveolar echinococcosis also occurs across the border in Kashmir, India [17]. Both cystic echinococcosis and schistosomiasis remain endemic in China [18], [19]. Among the serious protozoan infections, China and India (but mostly India) account for 70% of the world's cases of visceral leishmaniasis (kala-azar) [20] and, according to newer estimates, almost one in five deaths from malaria occurs in India [21]–[23].

**Table 2:** Neglected tropical diseases in China and India.

NTD	China [8]–[10]	India [11], [12]	Combined	Approximate percentage of global disease burden	Comment
**Parasitic infections**					
Ascariasis	86 million [9], [10], [13]	140 million [12], [13]	226 million	28% [14]	Based on 807 million cases globally [14]
Hookworm	39 million [9], [10], [13]	71 million [12], [13]	110 million	19% [14]	Based on 576 million cases globally [14]
Trichuriasis	29 million [9], [10], [13]	73 million [12], [13]	102 million	17% [14]	Based on 604 million cases globally [14]
Lymphatic filariasis	Disease eliminated [9], [10]	610 million requiring mass drug administration [12]	610 million people requiring mass drug administratin	43% [15]	Based on 1.4 billion people globally requiring mass drug administration [15]
Paragonimiasis	14–23 million [9], [10], [16]	Not determined	>14 million	60–99% [16]	Based on 23.2 million cases globally [16]
Clonorchiasis	12–13 million [9], [10], [16]	None	12–13 million	80–90% [16]	Based on 15.3 million cases globally [16]
Alveolar echinococcosis	16,629 [17]	Not determined	>16,000 cases	>91% [17]	Based on 18,235 cases globally [17]
Schistosomiasis	<700,000 [9], [10]	None	<700,000 [9], [10]	<1% [19]	Based on 207 million cases globally [19]
Visceral leishmaniasis	760 to 1500 cases (incidence) [20]	146,700 to 282,800 cases (incidence) [20]	147,460 to 284,300 cases [20]	71–72% [20]	Based on 0.2 to 0.4 million cases (incidence) [20]
Malaria deaths	<1,000 [21]	205,000 [22]	205,000	17% [23]	Calculated on the basis of 1.238 million malaria deaths globally [23]
**Bacterial and viral Infections**					
Tuberculosis deaths	47,000 [24]	300,000 [24]	347,000	35% [25]	Based on 990,000 deaths in HIV-negative people and 430,000 HIV-associated TB deaths globally [25]
Leprosy	2,468 registered cases [26]	83,187 registered cases [26]	85,655 registered cases	47% [26]	Based on more than 180,000 registered cases globally [26]
Trachoma	101,000 cases of active trachoma in WHO Western Pacific region [27]	196,000 cases of active trachoma in WHO South-East Asia region [27]	297,000	1% [27]	Based on 21.4 million cases globally [75]
Rabies	>2,000 cases [28]	20,000 cases [12]	22,000	40% [29]	Based on 55,000 deaths globally [29]
Japanese encephalitis	35,000–50,000 cases anually (8,000–10,000, incidence) [30], [31]	1,500–4,000 (incidence)^12,31^	Approx. 50,000 cases annually	>50% [30]	Based on statement made in Ref [30]
Dengue	6.5 million apparent cases 20.1 million inapparent cases 26.6 million cases total [32]–[34]	32.5 million apparent cases 99.7 million inapparent cases 132.2 million cases total [34]	158.8 million cases	41% [34]	Based on 390 million cases in 2010 [34]

Among the neglected bacterial infections, 35% of all deaths from tuberculosis occur in China and India [24], [25], and these two nations account for more than 40% of the world's leprosy cases [10], [12], [26]. Trachoma is still endemic in China and India [27]. Among the neglected viral infections, 40% of the global rabies deaths, mostly from canine rabies, occur in China and India [12], [28], [29]. Two flavivirus infections also stand out: China and India account for more than 50% of the global cases of Japanese encephalitis [12], [28], [31], and together these two nations account for more than 40% of the global burden of dengue with possibly more than 150 million cases annually [34]. Southern China (especially Guangdong Province) saw an increasing tendency for imported cases of dengue during the last decade [32], [33].

Under the leadership of the National Institute of Parasitic Diseases of the Chinese Centers for Disease Control and Prevention [35] and the Indian Council of Medical Research, enormous strides have been made in NTD control in China and India, respectively. For instance, in China lymphatic filariasis has been eliminated and the prevalence of schistosomiasis dramatically reduced over the last half of the 20^th^ century [8], [36], [37]. China has also been an important innovator for new anthelminthic drugs and diagnostics and for the development of OMICs technologies [38]–[41]. Similarly, India has made enormous strides in the eradication of guinea worm [42], and the control and elimination of leprosy and lymphatic filariasis [43]–[45]. Likewise, India has been an important innovator for new NTD therapeutics.

Despite these important gains, China and India are still faced with a devastating burden of disease resulting from parasitic, bacterial, and viral NTDs; and despite their rapidly growing economies these two nations still account for approximately 20–70% of the global burden from the major NTDs. In addition to their health impact, there is growing evidence that the NTDs trap people in poverty and thwart economic growth [7], so that the NTDs may represent a reason why China and India have not progressed further as emerging market economies.

Given the ability of scientists working in China and India to pursue advanced technologies and the financial and other resources made available to achieve these goals, there is every reason to believe that both China and India also possess the “scientific horsepower” and the resources to tackle their indigenous NTDs [46]. Solving the NTD problem in China and India has enormous potential dividends both in terms of reducing the global disease burden by up to 40% or more and simultaneously promoting economic growth and public health in Asia.

Controlling and eliminating NTDs in Asia will require a scale-up of mass drug administration, especially for the soil-transmitted helminth infections, lymphatic filariasis, food-borne trematode infections, and trachoma, while simultaneously conducting research and development for new drugs, diagnostics, and vaccines [8]. In parallel with these activities is an important yet underutilized opportunity for international cooperation and science diplomacy.

China and India share an extensive 2,200 mile border and an ancient history [47]. Most of that history has been peaceful, although a border war occurred in 1962 [46], [47]. In 2005, China and India partially resolved some key border differences in an agreement between Indian premier Manmohan Singh and Chinese premier Wen Jiabao, with additional commitments to promote joint economic cooperation and information technology [47]. Subsequently, key trade agreements were announced [48].

Ultimately, the huge disease burden from NTDs now shared between China and India creates an interesting opportunity to enhance scientific cooperation between these nations. For example, India might learn from China's historical successes at eliminating lymphatic filariasis, while China might benefit from India's recent experience in combating dengue and other arboviruses. Both nations could expand mass drug administration in order to reduce the number of cases of soil-transmitted helminth infections and trachoma, as well as to reduce a staggering burden of disease from tuberculosis. There also remains a high burden from malaria in India and in China's southwestern region. The fact that such a heavy burden of tropical diseases now occurs in two of the world's largest economies, each with top-ranked universities—Beijing, Tsinghua, and Fudan Universities in China and the Indian Institute of Technology, Centre for Cellular and Molecular Biology (CCMB), and All India Institute of Medical Sciences—and the recognized ability to produce nuclear technologies, indicates that the talent to achieve science diplomacy goals is already present. On the medical research front there are almost limitless opportunities to collaborate on new drugs, diagnostics, and vaccines for NTDs common to both nations [49].

In 2005 the governments of India, Nepal, and Bangladesh signed an important agreement at the World Health Assembly (Geneva) for mutual cooperation in order to eliminate visceral leishmaniasis from South Asia [50]. This agreement could be considered a landmark achievement in international science and medical diplomacy. We would urge that government leaders support China and India's major public health and research institutions devoted to NTDs and possibly look to that agreement as a starting point for identifying common ground and exploring opportunities for a Sino-Indian partnership on NTDs.
